# The Role of Interleukin-1-Receptor-Antagonist in Bladder Cancer Cell Migration and Invasion

**DOI:** 10.3390/ijms22115875

**Published:** 2021-05-30

**Authors:** Lisa Schneider, Junnan Liu, Cheng Zhang, Anca Azoitei, Sabine Meessen, Xi Zheng, Catharina Cremer, Christian Gorzelanny, Sybille Kempe-Gonzales, Cornelia Brunner, Felix Wezel, Christian Bolenz, Cagatay Gunes, Axel John

**Affiliations:** 1 Department of Urology, Ulm University Hospital, 89081 Ulm, Germany; Lisa.Schneider@uni-ulm.de (L.S.); liu.junnan88@outlook.com (J.L.); cheng.zhang@uni-ulm.de (C.Z.); anca.azoitei@uniklinik-ulm.de (A.A.); Sabine.Meessen@uniklinik-ulm.de (S.M.); xi.zheng@uniklinik-ulm.de (X.Z.); catharina.cremer@uni-ulm.de (C.C.); Felix.Wezel@uniklinik-ulm.de (F.W.); Christian.Bolenz@uniklinik-ulm.de (C.B.); axel.john@uniklinik-ulm.de (A.J.); 2 Department of Dermatology und Venerology, UKE, 20246 Hamburg, Germany; c.gorzelanny@uke.de; 3 Department of Otorhinolaryngology, Head and Neck Surgery, Ulm University Hospital, 89075 Ulm, Germany; sybille.kempegonzales@uniklinik-ulm.de (S.K.-G.); cornelia.brunner@uniklinik-ulm.de (C.B.)

**Keywords:** bladder cancer, cytokines, interleukin 1 receptor, IL1RA, invasion, migration

## Abstract

Background: The interleukin-1-receptor antagonist IL1RA (encoded by the *IL1RN* gene) is a potent competitive antagonist to interleukin-1 (IL1) and thereby is mainly involved in the regulation of inflammation. Previous data indicated a role of IL1RA in muscle-invasive urothelial carcinoma of the bladder (UCB) as well as an IL1-dependent decrease in tissue barrier function, potentially contributing to cancer cell invasion. Objective: Based on these observations, here we investigated the potential roles of IL1RA, IL1A, and IL1B in bladder cancer cell invasion in vitro. Methods: Cell culture, real-time impedance sensing, invasion assays (Boyden chamber, pig bladder model), qPCR, Western blot, ELISA, gene overexpression. Results: We observed a loss of IL1RA expression in invasive, high-grade bladder cancer cell lines T24, UMUC-3, and HT1197 while IL1RA expression was readily detectable in the immortalized UROtsa cells, the non-invasive bladder cancer cell line RT4, and in benign patient urothelium. Thus, we modified the invasive human bladder cancer cell line T24 to ectopically express IL1RA, and measured changes in cell migration/invasion using the xCELLigence Real-Time-Cell-Analysis (RTCA) system and the Boyden chamber assay. The real-time observation data showed a significant decrease of cell migration and invasion in T24 cells overexpressing IL1RA (T24-IL1RA), compared to cells harboring an empty vector (T24-EV). Concurrently, tumor cytokines, e.g., IL1B, attenuated the vascular endothelial barrier, which resulted in a reduction of the Cell Index (CI), an impedance-based dimensionless unit. This reduction could be reverted by the simultaneous incubation with IL1RA. Moreover, we used an ex vivo porcine organ culture system to evaluate cell invasion capacity and showed that T24-IL1RA cells showed significantly less invasive capacity compared to parental T24 cells or T24-EV. Conclusions: Taken together, our results indicate an inverse correlation between IL1RA expression and tumor cell invasive capacity and migration, suggesting that IL1RA plays a role in bladder carcinogenesis, while the exact mechanisms by which IL1RA influences tumor cells migration/invasion remain to be clarified in future studies. Furthermore, we confirmed that real-time impedance sensing and the porcine ex vivo organ culture methods are powerful tools to discover differences in cancer cell migration and invasion.

## 1. Introduction

Urothelial carcinoma of the urinary bladder (UCB) can be grossly subdivided into non-muscle invasive (NMIBC) and muscle-invasive tumors (MIBC). The overall rate of progression from NMIBC to MIBC lies between 20% and 30% [1]. A scoring system, based on clinical and pathologic criteria, aims to predict progression by subdividing NMIBC into low-, intermediate-, and high-risk tumors [2]. While low-risk tumors have a small likelihood of recurrence and progression, high-risk tumors, such as pT1 or carcinoma in situ (CIS), are known to become muscle-invasive in 50% of patients, if left untreated [3]. The most accurate important prognostic index is the current WHO 2016 histologic grading system, where a high tumor grade strongly correlates with progression to a muscle-invasive stage [4]. High throughput sequencing analyses provided a comprehensive molecular characterization of bladder cancer [5]. Additionally, alterations in molecular pathways have proven to be characteristic for either high- or low-risk disease. For example, FGFR3 mutations are commonly found in papillary, non-invasive, low-grade carcinomas [4]. A remaining task is to discover further molecules that are directly involved in tumor progression, with the hope of identifying potential therapeutic targets.

To identify such molecules, in vitro models are available to assess different aspects of malignancy in bladder cancer (BC) cell lines. Cell viability, migration, and invasion of cancer cells can be monitored using classic endpoint assays, such as the MTT and the Boyden chamber assays. However, a more recent electrophysiological method now enables real-time monitoring and objective quantification of the invasive potential of BC cells. Results obtained with the xCELLigence Real-Time Cell Analysis (RTCA) device have shown strong correlations with conventional endpoint methods mentioned above [6,7]. The higher sensitivity of RTCA methods allows for a better identification of new molecules involved in UCB progression and metastatic development [8].

One such possible molecule is the interleukin-1-receptor antagonist IL1RA (encoded by the *IL1RN* gene). It is a potent competitive antagonist to interleukin-1 (IL1) and mainly involved in regulating inflammation [9]. IL1RA was previously identified as an IL1 inhibitor in the supernatants of human cells [9]. This isoform now is referred to as secreted, sIL1RA. Besides the secreted isoform, additional IL1RA intracellular isoforms have been described in various cell types, including epithelial cells [10,11,12,13]. Although there is broad evidence showing that sIL1RA acts as a competitive inhibitor of IL1-alpha and -beta by binding to cell-surface receptors, there is scarce information on the role of the cytoplasmic IL1RA proteins [11]. Genetic polymorphisms in the *IL1RN* gene have been linked to an increased risk of developing gastric cancer [14], prostate cancer [15], and UCB [16]. A downregulation of *IL1RN* expression that coincides with the degree of malignancy has been observed in colorectal cancer [17], chronic myeloid leukemia [18], and in oral squamous cell carcinoma [19]. Immunohistochemical staining of UCB tissue samples has yielded similar results, finding significantly reduced IL1RA expression in MIBC samples compared to NMIBC and control samples [20].

Here we show that low expression of IL1RA correlates with a higher invasive capacity of bladder cancer cell lines and vice versa. Consequently, ectopic expression of IL1RA impairs migration and invasive capacities of aggressive UCB cells. Importantly, congruent results were observed in different experimental systems, such as RTCA analysis, Boyden chamber assays, and an ex vivo porcine organ culture model. Moreover, we show that recombinant IL1RA blocks IL1B-dependent endothelial barrier breakdown in the impedance-based assay. Taken together, the present data indicate that the IL1 family plays an important role in UCB metastatic progression.

## 2. Results

### 2.1. IL1RA Expression Is Downregulated in Invasive Bladder Cancer Cell Lines

**Figure 1:**
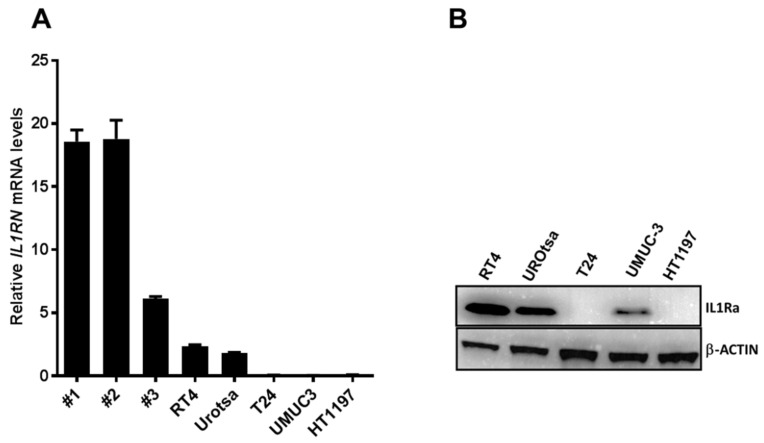
IL1RA expression is absent in invasive bladder cancer cell lines. (**A**) *IL1RN* mRNA levels in freshly isolated benign patient ureteral urothelium (#1, #2, #3 indicate individual sample numbers), and in non-invasive (RT4, UROtsa) and invasive (T24, UMUC-3, and HT1197) cell lines as determined by RT-qPCR. (**B**) Immunoblot results showing IL1RA protein levels in the indicated cell lines. Both the qPCR and the Western blot yielded only one specific band at the expected size. Please note that the qPCR primers were designed to amplify all potential *IL1RN* isoforms. Similarly, the antibody recognizes all potential IL1RA variants. Please note that primary human tissue samples could not be tested by Western blot due to low tissue availability.

We previously showed that IL1RA is highly expressed in the bladder urothelium, while its levels are downregulated in MIBC and low IL1RA levels are associated with aggressive disease [20]. In line with these observations, here we show that intrinsic *IL1RN* expression was highest in benign ureter epithelium (Samples #1, #2, and #3 in Figure 1A). *IL1RN* could also be detected, though at lower levels compared to the primary tissue samples, in the non-invasive RT4 cells and the immortalized benign cell line UROtsa, while *IL1RN* expression is lost/very low in invasive cell lines, such as T24, UMUC-3, and HT1197 (Figure 1A). These results were verified on the protein level via Western blot analysis. Both RT4 and UROtsa lysates showed a strong and clear band at the expected molecular weight of 20 kDa. Coinciding with their mRNA levels, IL1RA protein levels were low (UM-UC-3) or not detectable (T24, HT1197) (Figure 1B). In line with our initial hypothesis, these data suggest an inverse correlation between *IL1RN* expression and the invasion/migration capacity of urothelial cells.

### 2.2. Ectopic IL1RA Impairs T24 Cell Invasive and Migration Capacities

**Figure 2:**
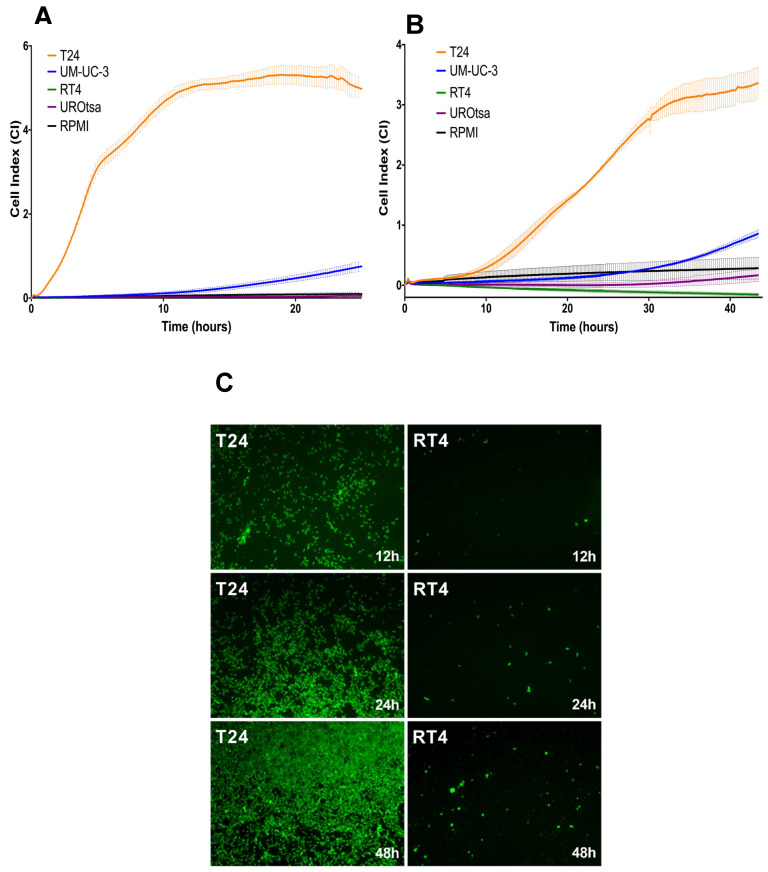
Evaluation of migration and invasion capacity of bladder cancer cell lines by RTCA and Boyden chamber assays. Migration (**A**) and invasion (**B**) capacities of bladder cancer cell lines as determined by RTCA impedance sensing. The cell index (CI) directly correlates to the migration and invasion capacity of cells (see Materials and Methods for experimental details). The experiment was performed three times, with quadruplicates of each experiment, and similar results were obtained. Depicted are the mean CI ± SD calculated from four replicates of each experiment (*n* = 3). (**C**) Modified Boyden chamber assay. Tumor cells stained with CellTracker™ Green CMFDA Dye migrate through a HUVEC-coated membrane. After 12, 24, and 48 h, T24 cells were detected in the lower well (green signal) at a much higher rate than RT4 cells. Representative immunofluorescence images of at least three independent experiments are shown.

We performed impedance-based real-time cell analysis (RTCA) to evaluate the migration and invasion capacities of the parental bladder cancer cell lines. Overall, the recorded cell index (CI) correlated with the reported migratory or invasive potential of the cancer cells while it was inversely connected to the respective IL1RA expression levels. IL1RA negative T24 cells reached the highest CI values, both in the migration (RTCA w/o Matrigel) and invasion (RTCA with Matrigel) setting. Interestingly, a moderate CI increase could be observed in UMUC-3 cells, in line with the observed IL1RA expression at the protein level (See Figure 1B). T24 cells harbour the highest migration and invasion potential, as indicated by high CI while IL1RA-positive cell lines UROtsa and RT4 cells consistently showed a low CI, similar to the negative control (RPMI cell culture medium only). The results obtained with RT4 and T24 cells were confirmed in a modified Boyden chamber assay where the surface was covered with a HUVEC monolayer (Figure 2C).

After identifying *IL1RN* as a potential modulator of the invasive capacity of cancer cells, we used ectopic expression in T24 cells to address the question of whether IL1RA expression itself is sufficient to impair the migration and invasion capacity of BC cells. The T24 cells were infected with retroviral particles containing an *IL1RN* cDNA, which we amplified from RT4 cells and cloned into the pBABE-Puro retroviral vector to generate IL1RA-expressing T24 cells (T24-IL1RA). RT-qPCR confirmed a significant five-fold increase in *IL1RN* expression in infected T24-IL1RA cells compared to parental T24 and mock-infected T24 (T24-EV: pBabe-Puro empty vector) cells. Successful overexpression at the protein level was confirmed by Western blot analysis, indicating a strong and clear signal for IL1RA expression.

**Figure 3:**
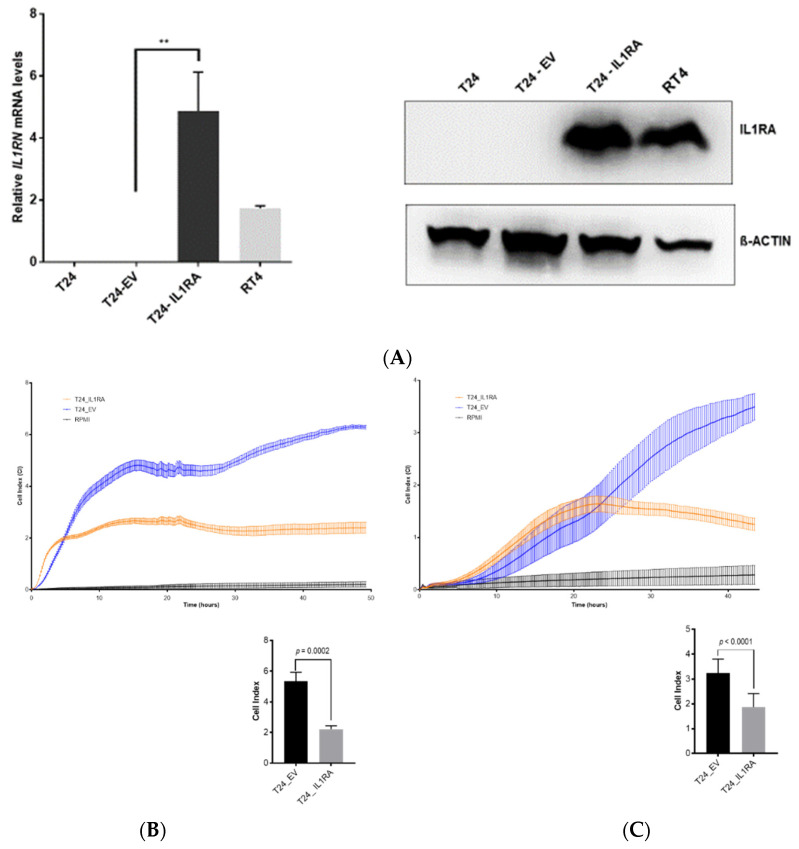

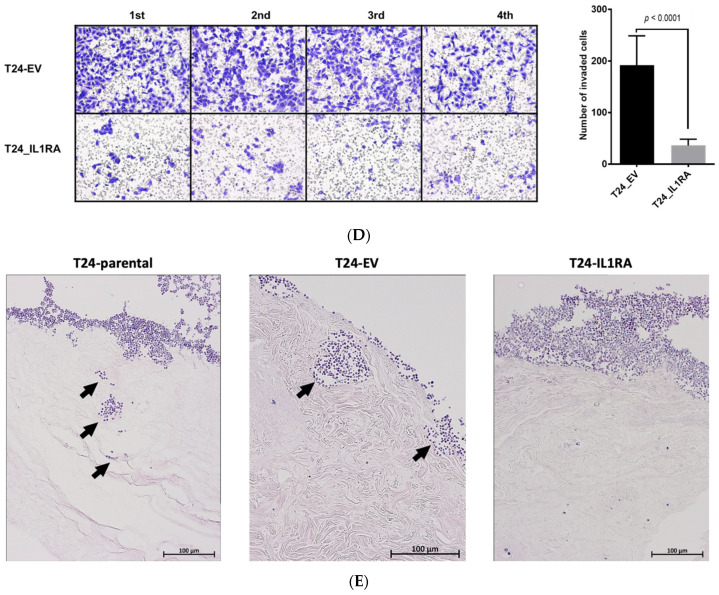
Ectopic IL1RA expression impairs migration and invasion capacity of invasive T24 cell line in RTCA experiments. (**A**) Ectopic *IL1RN* expression in invasive T24 cells. (Left) RT-qPCR showing the ectopic *IL1RN* expression in infected T24-IL1RA cells (T24-pBabe-IL1RN) compared to parental T24 and mock-infected empty vector T24 (T24-pBabe-Puro) (** indicates *p* = 0.026). *IL1RN* expression in RT4 cells served as a positive control. (Right) Western blot results confirmed the overexpression of IL1RA. T24-IL1RA exhibit a strong IL1RA signal, with an intensity similar to the positive control RT4 cells. T24-negative control cells (T24 parental and T24-EV) showed no signal for IL1RA. β-ACTIN served as a loading control. (**B**,**C**) The graphs show migration (left) and invasion (right) capacities of T24-IL1RA (T24-pBabe IL1RN) compared to T24-EV (T24-pBabe-Puro). Wells containing serum-free media (RPMI) served as control. In the migration setting (**B**), CI values of T24-IL1RA are consistently lower than those of T24 empty vector. In the invasion setting (**C**), the difference in CI clearly manifests after 30 h. The graphs show the quantification of the cell index (mean) at the 40 h time-point. The experiment was performed two times (*n* = 2) in the migration setting and three times in the invasion setting, with quadruplicates of each experiment. Depicted are the mean CI ± SD calculated from quadruplicates of one experiment. (**D**) Ectopic *IL1RN* expression impairs migration and invasion capacity of invasive UCB cell lines as determined by the Boyden chamber assay. Four independent repeat experiments (1st, 2nd, 3rd, and 4th) were performed to determine the impact of IL1RA on cell invasion through Matrigel after an incubation period of 48 h (left). The results were evaluated quantitatively by manually counting the cells in five randomly selected membrane areas using the image processing program ImageJ (Version 1.51). The average number of invaded cells of T24-IL1RA is significantly lower compared to T24-EV (right). (**E**) Ectopic *IL1RN* expression impairs the invasion capacity of the T24 cells in the porcine bladder organ culture model. Representative pictures are shown indicating invasion (black arrows) of the parental T24 (left) and T24 cells containing the empty vector (middle) into the porcine lamina propria while invasion was completely absent with the T24-IL1RA cells (right). Images were taken with the microscope Axio Imager 2 (Zeiss, Oberkochen) at 200× magnification.

The migration and invasion capacity of the T24-IL1RA cells was determined by the impedance-based RTCA method, the Boyden chamber assay, and an ex vivo organ culture invasion assay (Figure 3B–E). The RTCA assay showed a significant decrease of cell migration and invasion of T24-IL1RA compared to T24-EV cells (Figure 3B,C). Since the infected T24-IL1RA and T24-EV cells were kept under permanent puromycin selection, the parental T24 cells were not tested in the functional experiments. In the migration setting (RTCA w/o Matrigel), a significant difference in cell index (CI) was already obvious at 10 h and stabilized after approximately 45 h (Figure 3B). The onset of invasion was delayed in the invasion setting (RTCA with Matrigel), with significant differences in CI clearly manifesting after 30 h (Figure 3C). These data indicate that the RTCA allows quantification of migration and invasion of cells over a longer time period.

Consistent with the RTCA results (CI dropped from 3.6 with T24-EV to 1 with T24-IL1RN cells), the overexpression of *IL1RN* strongly impaired the invasion of T24-IL1RA cells through a Matrigel layer in the Boyden chamber experiment (Figure 3D). Both assays thereby indicate an inverse correlation between *IL1RN* expression and tumor cell invasive capacity and migration.

In addition to the in vitro cell culture experiments, we evaluated the impact of IL1RA on the invasion capacity of T24 cells in an ex vivo organ culture model. For this purpose, we used the porcine bladder system [21,22], with some modifications [23] (see also Materials and Methods for detailed protocol). Importantly, we observed that ectopic IL1RA attenuated the invasive potential of T24 cells, strongly supporting the results obtained by the Boyden chamber assay and RTCA experiments.

### 2.3. IL1RA Impairs IL1B-Induced Tumor Cell Invasion through the HUVEC Monolayer

**Figure 4:**
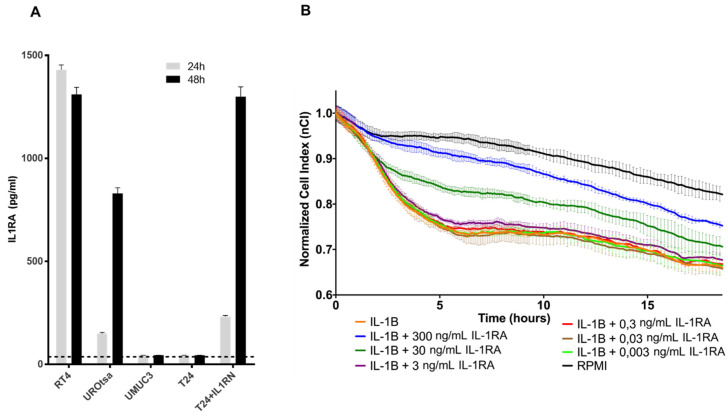
Recombinant IL1RA impairs IL1B-induced breakdown of HUVEC monolayer barrier function. (**A**) Secreted IL1RA determination. Secreted IL1RA in the supernatant (SN) of the indicated samples as determined by ELISA. The dashed line indicates the background of the ELISA signal. The results are mean values of three independent experiments. Considerable concentrations of IL1RA could be detected in the SN of RT4, UROtsa, and T24 + ILRN cells. (**B**) IL1B-induced breakdown of HUVEC monolayer barrier function is attenuated by IL1RA addition in a dose-dependent manner. The HUVEC monolayer is exposed to IL1B (1 ng/mL) or a combination of IL1B with varying concentrations of IL1RA (0.003 ng/mL, 0.03 ng/mL, 0.3 ng/mL, 3 ng/mL, 30 ng/mL, 300 ng/mL). HUVECs stimulated with serum-free media (RPMI) served as control. The CI was normalized to a time-point after cytokine addition. Depicted is the mean of normalized CI ± SD derived from four replicates in one experiment. The experiment was performed three times (*n* = 3) with similar results.

The IL1 family consists of two agonists, IL1A and IL1B, and the IL1-receptor antagonist, IL1RA. Their action is mainly studied within the context of inflammation, and the balance between IL1 and IL1RA plays an important role in their physiological action via the IL1-receptor [11]. Interestingly, it was shown that the proinflammatory cytokines IL1A and IL1B are most probably also related to bladder cancer invasion. We have therefore analyzed the impact of IL1A and IL1B on the invasive and migration capacities of T24 cells using impedance assay and the Boyden chamber assay. Both IL1A and IL1B provided comparable induction of migration/invasion capacities in both assays (Supplementary Figure S1). Nevertheless, the role of different IL1 subtypes in bladder cancer is controversial, with evidence pointing to a more prominent role of IL1B in comparison to IL1A [24,25,26]. Therefore, subsequent experiments were only performed with IL1B. To evaluate the potential interplay between IL1RA and IL1B in the context of bladder cancer cell invasion, we first identified the release of sIL1RA in the supernatant of RT4 and UROtsa cells (endogenous IL1RA) as well as of T24-IL1RA (ectopic IL1RA). In contrast, supernatant of parental T24 and UMUC cells was negative for IL1RA altogether, in line with the qPCR results shown in Figure 1 (Figure 1A and Figure 4A). The potential impact of IL1B on the functionality of physiological barriers as well as its reversal by IL1RA was evaluated in the next set of experiments. For this purpose, HUVECs were seeded in the wells of the RTCA device, thus mimicking the vascular endothelium (Figure 4B).

The CI of the HUVEC monolayer was then continuously monitored, serving as a surrogate marker for the endothelial barrier function and integrity. Tumor-derived cytokines can cause barrier breakdown, potentially by impairing tight junctions between neighbouring cells [27]. HUVECs formed a high-resistance monolayer on top of the microelectrodes after a period of 24 h. Exposure to recombinant IL1B (1 ng/mL) caused a characteristic breakdown pattern, with the normalized Cell Index (nCI) value dropping by 0.3 units (Figure 4B). Addition of recombinant IL1RA attenuated the endothelial breakdown in a dose-dependent manner. This effect could be seen clearly at IL1RA concentrations between 30 ng/mL and 300 ng/mL (Figure 4B). Taken together, these data indicate that IL1RA seems to antagonize the IL1B-induced endothelial barrier breakdown in a HUVEC monolayer model.

## 3. Discussion

Considerable progress in cancer therapy has been documented over the last years; however, patients with metastatic UCB still suffer from a lack of curative therapies [28]. For the colonization of distant sites, cancer cells must leave the primary tumor, then enter and exit the blood circulation. This process of intravasation and extravasation is termed trans-endothelial migration (TEM) and the vascular endothelial layer represents a critical barrier for tumor cells [29]. In contrast to the usually leaky and dysfunctional tumor neo-vessels, the endothelial surface in distant organs is intact and it is highly probable that TEM is an active process promoted by cytokines, growth factors, and vascular permeability regulators. In response to these environmental stimuli, ECs adapt to the new conditions and undergo activation (endothelial cell activation, ECA), which in turn creates proinflammatory and procoagulatory intravascular conditions. ECA enables multiple heterotypic adhesive interactions of tumor cells with platelets, leucocytes, and the endothelium, facilitating subsequent tumor cell docking on the endothelium and extravasation [30,31,32]. Therapeutic intervention in this intravascular multicellular crosstalk and the accompanying local hypercoagulation, for example, using low molecular heparins, has emerged as a promising option for attenuating the risk of venous thrombo-embolism and metastatic dissemination [33,34].

In the present study, we investigated the role of IL1RA in the context of ECA and as a potential modulator of migration and tissue invasion in UCB. We show that low *IL1RN* mRNA levels correlate with increased migration and tissue invasion capacities of bladder cancer cell lines. Conversely, *IL1RN* mRNA levels were higher in semi-benign UROtsa and non-invasive RT4 cell lines as well as in primary epithelial cells derived from apparently healthy ureter tissue, supporting the idea that IL1RA may impair invasiveness of bladder cancer cells. Notably, independent experimental approaches (Boyden chamber, impedance measurement, and ex vivo porcine invasion assay) provided congruent results, confirming the impact of ectopic IL1RA on cell invasiveness and migration. While the established Boyden chamber is a simple to perform end-point technique to evaluate the migration and invasion capacities, impedance sensing allows continuous real-time monitoring of the integrity and concomitant barrier function of cell layers, and thus represents a useful adjunct to classical end-point assays. Here, we combined the xCELLigence impedance sensing system with surface-coating of the well with Matrigel or a HUVEC monolayer. In both approaches, ectopic IL1RA in the highly invasive T24 cells lead to a markedly reduced invasive potential and attenuated cell migration compared to the parental cell line or cells transfected with an empty vector. Importantly, we also used the ex vivo porcine bladder organ culture model [35], which allows the evaluation of tumor cell invasiveness under semi in vivo conditions [21]. Again, IL1RA overexpression led to a significant loss of invasive potential of the tested cells, strongly supporting the conclusion that IL1RA impairs the invasive and migration capacities of bladder cancer cells. However, while overexpression of *IL1RN* in T24 cells attenuated invasiveness, shRNA-mediated knockdown of *IL1RN* in the non-invasive RT4 cells did not result in an increased invasiveness using RTCA (not shown). It remains to be elucidated whether IL1RA acts in concert with additional factors whose altered expression and/or functionality cooperates with IL1RA for efficient modulation of cell invasiveness.

The functions of IL1RA were mainly studied in the context of IL1 activity, where secreted IL1RA (sIL1RA) antagonizes the effects of IL1A or IL1B through inhibition of IL1 binding to surface receptors. In addition to sIL1RA, three intracellular IL1RA (icIL1RA) isoforms have been described [10,12]. The functions of the icIL1RA isoforms are still not clear. In line with our previous report [20], the present data show that low IL1RA levels in bladder cancer cell lines correlate with their increased invasive potential. On the other hand, we find that the endogenous IL1RA (RT4 and UROtsa) as well as the ectopic IL1RA (T24-IL1RA) can be measured in the supernatant of cells, indicating that it is secreted. At this time-point, we do not have a conclusive explanation how exactly IL1RA impairs invasion and migration capacity of the cells. The vasculature is one of the first obstacles to overcome for tumor cells during invasion of distant tissues. In our investigation, we used a HUVEC monolayer to fully cover the wells of the impedance sensing system as a model for the vascular endothelium. Addition of IL1B led to a decline in HUVEC barrier function, which could be reversed by the addition of IL1RA in a dose-dependent manner (Figure 4B). Thus, IL1 might be an important promoter of TEM, and external (secreted) IL1RA acts in an antagonistic manner impairing IL1B TEM activity.

Taken together, our data are in line with our previous report showing a reduced expression of IL1RA in human bladder cancer samples in comparison to healthy urothelium. Importantly, we reported that reduced IL1RA expression correlated with adverse pathologic characteristics [20]. Furthermore, polymorphisms in the *IL1RN* gene were associated with recurrence after BCG immunotherapy and susceptibility to bladder cancer [36]. More aspects linked to the IL1 pathway have so far been described in UCB, such as IL1B-induced cisplatin-resistance by up-regulation of Aldo-keto reductase 1C1 [24], IL1 dependent intra-tumoral androgen receptor (AR) signaling, T-cell attraction, and recruitment of tumor-associated fibroblasts [26]. We think that molecular subtyping of bladder cancer will become an element of clinical routine in the upcoming years. For instance, it was shown that patients with luminal MIBC do not appear to derive much clinical benefit from neoadjuvant chemotherapy compared to basal type MIBC. On the contrary, patients with luminal tumors that are infiltrated with stromal cells seem to be sensitive to immune checkpoint inhibitors such as anti PD-L1. Since the IL1 axis is a strong regulator of inflammatory response, one might speculate that loss of IL1RA in high-grade bladder cancer might play a greater role in those tumors with marked immune cell infiltration. Finding a correlation between IL1/IL1RA expression and molecular subtypes of bladder cancer would be an interesting topic for further research.

In summary, IL1RA might have an anti-tumorigenic role in bladder cancer by attenuating tumor cell migration and invasion while preserving the integrity of vascular endothelial barriers. As impacts on migration and invasion capability were observed in the absence of a vascular endothelial layer, it is tempting to speculate that IL1RA may also contribute to cells’ invasive capacity by a yet unknown intracellular mechanism. It is also conceivable that IL1RA acts in an autocrine manner to modulate the cells’ invasion and migration capacity. While the exact molecular pathways and downstream targets remain to be uncovered, our study was able to show the possible relevance of IL1RA for tumor progression in UCB using a variety of experimental methods, including an ex vivo organ culture model and in vitro cell culture experiments. Further in vivo trials are necessary to evaluate the potential of IL1RA as a potential therapeutic target in bladder cancer patients.

## 4. Materials and Methods

### 4.1. Cell Lines and Primary Human Urothelium

The human UCB cell lines T24, UM-UC3, and RT4 were obtained from the European Collection of Authenticated Cell Cultures (ECACC). The simian virus 40 (SV40) large T antigen immortalized UROtsa cell line served as a model for benign urothelium (gift of Dr. Phillip Erben, PhD, Mannheim). All cell lines were kept in RPMI 1640 medium (ThermoFischer Scientific, Waltham, MA, USA), supplemented with 10% fetal calf serum (FCS) (Sigma-Aldrich, St. Louis, MO, USA) and 1% penicillin/streptomycin (Pan Biotech, Aidenbach, Germany). The medium for UROtsa cells was additionally supplemented with 1% GlutaMAX (ThermoFischer Scientific, Waltham, MA, USA) and 1% non-essential amino acids (MerckMillipor, Darmstadt, Germany). Human umbilical vein endothelial cells (HUVEC) were obtained from umbilical cord veins as described previously [37] and grown in culture medium composed of two-thirds M199 supplemented with 10% heat-inactivated fetal calf serum, 1% antibiotics (penicillin and streptomycin), and one third EGM-2 (Lonza, Basel, Switzerland). HUVECs were maintained at 37 °C, 5% CO_2_. Cells were cultured at 37 °C in a humid atmosphere with 5% CO_2_. Primary human urothelial cells were prepared from benign ureter tissue of patients who underwent nephrectomy, with informed consent. Briefly, tissues were placed on ice directly after surgery and treated with stripper medium (500 mL HBSS without Ca^2+^Mg^2+^, Gibco 14170-088, 10 mM Hepes, Gibco 15630-056, and 0.1% EDTA) overnight. Epithelial cells were scratched and processed for RNA isolation immediately. The study was approved by the local Ethics Committee (ethical approval number: 239/18).

### 4.2. Cloning of IL1RN into the pBabe-Puro Plasmid Vector and Ectopic Expression in T24 Cells

A PCR product encoding the *IL1RN* gene was amplified from RT4 cDNA using the Phusion High-Fidelity PCR Kit (ThermoFischer Scientific, Waltham, MA, USA). The cDNA was generated by reverse transcription (RT) using 1 μg total RNA from RT4 cell cells with the GoScript^TM^ Reverse Transcription System (Promega, Fitchburg, WI, USA) in a 20 µL reaction mixture. The PCR reaction was conducted with 5 µL of the RT reaction and the cloning primers (forward primer: 5′-CGATGCGGATCCGAGGCCCTCCCCATGGCTTTAG-3′ and reverse primer 5′-CGATGCGTCGACGGCAGTACTACTCGTCCTCC-3′), which included BamHI and SalI restriction enzyme recognition sites, respectively. Cycling conditions: initial denaturation at 98 °C for 30 s, 25 cycles of 98 °C for 10 s, 55 °C for 30 s, followed by a final extension at 72 °C for 10 min. The PCR product was run on an agarose gel, followed by gel purification with the QIAquick^®^ Gel Extraction Kit (QIAGEN, Hilden, Germany). Both the expression vector pBabe-Puro and the purified PCR product were digested with the restriction enzymes BamHI-HF^®^ and SalI-HF^®^ (New England Biolabs, Ipswich, MA, USA) and ligated using Quick Ligase (New England Biolabs, Ipswich, MA, USA). The ligation mix was transformed into Stbl 3 chemically competent *Escherichia coli* (ThermoFischer Scientific, Waltham, MA, USA) and the plasmid DNA was isolated from a single bacterial colony with the PureYield™ Plasmid Miniprep System (Promega, Fitchburg, MA, USA). The cloning of IL1RN was validated by Sanger sequencing using pBABE-5′ or pBABE-3′ sequencing primers (Eurofins, Ebersberg, Germany). Plasmid DNA was amplified with the NucleoBond^®^ Xtra Midi/Maxi Kit (Macherey-Nagel, Düren, Germany). For virus production, HEK293 cells were cotransfected with the *IL1RN* expression vector, the pCMV-Gag-Pol and pCMV-VSV-G plasmids. The virus was harvested after 48 h, filtered twice through a 0.45 µm filter (Sarstedt, Nürnbrecht, Germany), and used to infect T24 cells. The infected target cells were selected with 1 μg/mL Puromycin (ThermoFischer Scientific, Waltham, MA, USA) for 7 days and permanent selection was continued with 0.5 μg/mL Puromycin.

### 4.3. Determination of IL1RA by ELISA

An amount of 1.5 × 10^7^ cells were seeded in T25 flasks (Sarstedt, Nürnbrecht, Germany) to achieve full confluency and to minimize effects by cell numbers due to different proliferation capacities of the cells. An amount of 1 mL supernatant (SN) was transferred into Eppendorf tubes (E-tubes) at 24 h and 48 h post-plating and immediately placed on ice for 2 min. Samples were then centrifuged at 1400 rpm for 10 min to pellet potential contaminating cells and 800 µL of the clean SN were transferred into new E-tubes and stored in −80 °C until use by ELISA. The ELISA was determined according to the protocol provided by the supplier, using 100 µL of the SN. The standard curve was determined by the recombinant IL1RA provided with the kit. The absorbance of the samples was read at 450 nm.

### 4.4. Quantitative Real-Time PCR (RT-qPCR)

Cells were harvested and total RNA was isolated with the RNeasy^®^ Mini Kit (QIAGEN, Hilden, Germany). Reverse transcription was performed with the GoScript^TM^ Reverse Transcription System (Promega, Fitchburg, MA, USA) using 100 ng total RNA. Diluted (1:10) cDNA was incubated with IL1RN-specific primers (IL1RN-F1: 5′-TGTTCCCATTCTTGCATGGC and IL1RN-R1: 5′-GCAGCATGGAGGCTGGTCAG) and the iTaq™ Universal SYBR^®^ Green Supermix (Bio-Rad, Hercules, CA, USA). The Viia™ 7 Real-Time PCR-System (Applied Biosystems, Foster City, CA, USA) was used for quantitative real-time PCR (RT-qPCR) of *IL1RN* and *GAPDH* [38]. The following conditions were used for quantitative real-time PCR: 1 × 50 °C for 2 min; 1 × 95 °C for 10 min; 40 × 95 °C for 15 s and 60 °C for 1 min, followed by 1 × 95 °C for 15 s, 1 × 60 °C for 1 min, 1 × 95 °C for 30 s, and 1 × 60 °C for 15 s. ΔCT-values were calculated using *GAPDH* as reference values to reflect the relative mRNA level in the BC cell lines. RT-qPCR results were obtained from three individual experiments, each performed in triplicate.

### 4.5. Western Blot

Cells were washed in DPBS (ThermoFischer Scientific, Waltham, MA, USA) and detached with trypsin (Pan Biotech, Aidenbach, Germany). The cell pellet was resuspended in RIPA buffer (Sigma-Aldrich, St. Louis, MO, USA) containing phosphatase and protease inhibitors (Roche Applied Science, Penzberg, Germany). Cells were mechanically destroyed, and the cell lysate solution was centrifuged at 14,000 rpm for 40 min at 4 °C. The protein concentration was determined by Bicinchoninic acid (BCA) assay. The protein amount was adjusted to 20 μg and the sample volume brought up to 20 μL with RIPA buffer. Additionally, 4 μL of 4× Laemmli Buffer (Bio-Rad, Hercules, CA, USA) with 5% 2-mercaptoethanol (Sigma-Aldrich, St. Louis, MO, USA) were added to each sample. The proteins were denatured at 96 °C for 10 min after being separated on a 10% acrylamide gel (Bio-Rad, Hercules, CA, USA) at 80 V for 2 h. The proteins were transferred to a PVDF membrane (Merck, Darmstadt, Germany) via electroblotting at 300 mA for 2 h. The membrane was blocked in TBS-Tween 20 (TBS-T) containing 5% skim milk for 45 min, followed by incubation with a primary monoclonal antibody overnight at 4 °C in TBS-T containing 5% skim milk (Sigma-Aldrich, St. Louis, MO, USA). The following primary antibodies were used: rabbit anti-IL1RA (1:500; Sigma-Aldrich, St. Louis, MO, USA, HPA001482) and mouse anti-β-actin (1:10,000; Sigma-Aldrich, St. Louis, MO, USA, A2228). Membranes were washed three times in TBS-T and subsequently incubated with an anti-rabbit/anti-mouse horseradish peroxidase (HRP)-linked secondary antibody (1:2000; Cell Signaling Technology, Danvers, MA, USA) for 2 h at room temperature. Membranes were then washed three times in TBS-T, incubated with AceGlow^TM^ chemiluminescence substrate (VWR, Radnor, PA, USA), and antibody signals were visualized by chemiluminescence detection (Fusion Fx, Vilbert Lourmat, Collégien, France).

### 4.6. Classical Boyden Chamber Invasion Assay

Cultures were split to reach 80% confluency and then starved with serum-free RPMI 1640 for 24 h. Pore culture inserts (8 μm; Sarstedt, Nürnbrecht, Germany) were placed into a 24-well plate (Sarstedt, Nürnbrecht, Germany) and subsequently coated with 100 μL pre-thawed Matrigel (250 μg/mL) (Corning Incorporated, Corning, NY, USA). The plates were left to incubate for 30 min at 37 °C. The bottom wells were filled with 800 μL RPMI 1640 containing 10% FCS as chemoattractant. Cells were washed with DPBS, detached with trypsin, and counted with a Neubauer hemocytometer. The cell suspension concentration was adjusted to 1 × 10^5^ cells/mL and 100 μL of cell suspension was added to each well and spread evenly by means of soft shaking. The plate was incubated at 37 °C under normal culture conditions. After 48 h, the inserts were removed, washed with DPBS, fixed with 500 μL 5% glutaraldehyde solution (Sigma-Aldrich, St. Louis, MO, USA) for 30 min, washed with DPBS, stained with 500 μL 0.2% crystal violet solution (Sigma-Aldrich, St. Louis, MO, USA) for 30 min, and rinsed with tap water. A cotton swab was used to remove non-migrated cells from the upper side of the insert membrane. The membranes were dried overnight and were then cut from the insert using a scalpel. Membranes were mounted face-down on a microscope slide with Entellan^®^ (Merck, Darmstadt, Germany) and sealed with a cover-slip. The positively stained cells were evaluated for each membrane by taking pictures of five random fields at 10× magnification and counting the cells manually using “ImageJ”. The results of the Boyden chamber experiments were obtained from four biological replicates.

### 4.7. Modified Boyden Chamber Invasion Assay

To simulate interactions of tumor cells with the vascular endothelium, the Boyden chamber experiment was modified in a set of experiments. Wells with 8 µm pores were coated with 100 µL 0.1% gelatin; 2 × 10^5^ HUVECs were seeded and wells incubated for 48 h until a confluent monolayer was obtained as confirmed by microscopy. Bladder cancer cells (RT4 or T24) were stained with CellTracker™ Green CMFDA Dye (Invitrogen, Carlsbad, CA, USA) according to the instructions of the manufacturer and kept in starvation medium for 24 h. Full growth medium supplemented with FCS as a chemoattractant was put in the lower part of the transwell while 1 × 10^5^ stained and starved RT4 or T24 cells were seeded unto the HUVEC layer. After 18 h incubation, the membrane of the lower part of the well containing the migrated tumor cells was cut, washed, fixed, and prepared for further immunofluorescence imaging.

### 4.8. Real-Time Impedance Sensing for Migration, Invasion, and Endothelial Barrier Function

Cell invasion and migration was continuously monitored using the impedance-based measuring device xCELLigence RTCA DP instrument (ACEA Biosciences, San Diego, CA, USA). The CIM-Plate 16 (ACEA Biosciences, San Diego, CA, USA) contains 16 wells that are divided into upper and lower chambers by a microporous polyethylene terephthalate (PET) membrane with 8 µm pores, allowing cells to adhere to microelectrodes on the underside of the membrane [39]. The number of adherent cells causes a change in impedance that is reflected by an increase in Cell Index (CI). The CI directly correlates with the cells’ migratory potential [7,39,40]. When monitoring cell invasion, the upper chamber was additionally coated with 250 μg/mL pre-thawed Matrigel (Corning Incorporated, Corning, NY, USA) diluted in cold, serum-free RPMI 1640 and incubated for 4 h at 37 °C. Under these conditions, CI directly correlates with the cells’ invasive potential.

Initially, the plate was prepared by coating the electrodes with 1% gelatin in DPBS followed by an incubation period of 45 min under the tissue culture hood. The gelatin was removed, and the electrodes were washed once with sterile water. The lower chamber was filled with RPMI 1640 containing 10% FCS as a chemoattractant for migrating/invading cells. The upper chamber was filled with serum-free RPMI. The plate was left to equilibrate at 37 °C for 1 h in the RTCA DP-Analyzer.

Cells were passaged two days prior and starved one day prior to the experiment, reaching a final confluency of 70–80%. During plate equilibration, cells were washed with DPBS, detached with trypsin, and counted with a Neubauer hemocytometer. Cells were centrifuged for 3 min at 1200 rpm at 24 °C. The cell pellet was resuspended in serum-free RPMI to obtain a concentration of 4 × 10^5^ cells/mL.

After plate equilibration, background measurements were recorded with the RTCA DP device. Subsequently, 100 μL of the cell suspension was carefully added to each well of the upper chamber to avoid bubble formation. The CIM-Plate 16 was placed in the RTCA DP-Analyzer and the CI was monitored over 48 h at 10–15 min interval measurements. Cell migration experiments were repeated twice and cell invasion experiments were repeated three times, both in quadruplicate.

The xCELLigence RTCA DP instrument (ACEA Biosciences, San Diego, CA, USA) was also used with the E-Plate 16 to assess the integrity of a HUVEC monolayer in response to treatment with IL1B and IL1RA. The electrodes were coated with 1% gelatin in DPBS and left to incubate at 37 °C for 1 h. The gelatin was removed, and the electrodes were washed once with sterile water. Each well was filled with HUVEC medium and incubated at 37 °C for 1 h for plate equilibration and background measurement. HUVECs with a maximum passage number of 5 were then seeded in each well at a seeding density of 30,000 cells/ well. The E-Plate 16 was left under the tissue culture hood to equilibrate for 30 min and then placed in the RTCA DP-Analyzer to begin with the impedance readings. The HUVECs were left to attach to the electrodes and form a monolayer within 20–24 h. This could be confirmed by a leveling out of the CI. IL1B (25 μg/mL; R&D systems, Wiesbaden, Germany) was diluted in RPMI to a final concentration of 1 ng/mL. IL1RA (100 μg/mL; R&D systems, Wiesbaden, Germany) was diluted in RPMI 1640 to final concentrations of 0.3 ng/mL, 3 ng/mL, 30 ng/mL, or 300 ng/mL. Half of the HUVEC medium was removed from each well and IL1B and IL1RA suspensions were added to each well. The E-Plate 16 was placed back into the RTCA device and the impedance readings were continued. The CI was monitored for another 20–24 h. For analysis, the CI was normalized to a time-point after IL1B and IL1RA addition. The experiment was repeated three times in quadruplicate.

### 4.9. Porcine Bladder Invasion Model

Porcine bladder was supplied by a local abattoir (Ulmer Fleisch GmbH, Ulm, Germany) and kept on ice during transport. The porcine bladder was cut open and washed three times in DPBS containing 10% penicillin/streptomycin. It was placed in 0.5% dispase II solution (Sigma-Aldrich, St. Louis, MO, USA) overnight at 4 °C to detach the urothelial cells from the basement membrane. The next day, the urothelium was removed with the blunt end of a scalpel and any loose connective tissue was removed with sterile scissors. The porcine bladder was cut into 0.5 cm^2^ pieces and each piece was placed onto a 70 μm cell strainer placed inside a 6-well plate containing 5.5 mL Waymouth’s medium (ThermoFischer Scientific, Waltham, MA, USA). The medium only covered the underside of the stromal tissue pieces, leading to an air-liquid interface. Silicon O-rings (diameter = 5 mm) were placed on the deepithelialized surface of each tissue piece. The porcine bladder was kept under normal culture conditions overnight. The next day, target cells were harvested and the seeding number adjusted to 1–1.5 × 10^6^ cells per tissue piece. The cell suspension was pipetted into the area enclosed by the silicon O-ring, ensuring that most cells would stay in contact with the stroma. The medium of the 6-well plate was changed to Keratinocyte-SFM (1X) (ThermoFischer Scientific, Waltham, MA, USA). After two days, the medium was changed to RPMI 1640 media, supplemented with 10% fetal calf serum (FCS), 1% penicillin/streptomycin, 1% GlutaMAX, and 1% non-essential amino acids. The tissue pieces were kept under normal culture conditions. The wells were washed with DPBS containing 10% penicillin/streptomycin and the medium was changed on alternate days. After two weeks, the tissue was removed and fixed in 10% formalin solution. Samples were dehydrated and embedded in paraffin wax. Vertical tissue cross-sections (3 μm) were dried overnight and stained with hematoxylin and eosin (Waldeck, Münster, Germany) for histological evaluation. Pictures were taken with the Axio Imager M2 microscope and Axiocam 105 color (Zeiss, Oberkochen, Germany).

### 4.10. Statistical Analysis

Results were expressed as mean ± SD. Statistical significance was determined by comparing values using an unpaired two-tailed *t* test. All statistical analyses were carried out using GraphPad Prism 7.0 (GraphPad Software, Inc., La Jolla, CA, USA).

## Data Availability

Not applicable.

## Supplementary Materials

The following are available online at https://www.mdpi.com/article/10.3390/ijms22115875/s1.
