# Supplementary material for: The Role of Interleukin-1-Receptor-Antagonist in Bladder Cancer Cell Migration and Invasion
Source: Int J Mol Sci. 2021 May 30;22(11):5875. doi: 10.3390/ijms22115875 (PMC8198563; doi:10.3390/ijms22115875)
Supplement: Supplementary file 1 [file ijms-22-05875-s001.zip › ijms-1231167-supplementary.pdf]

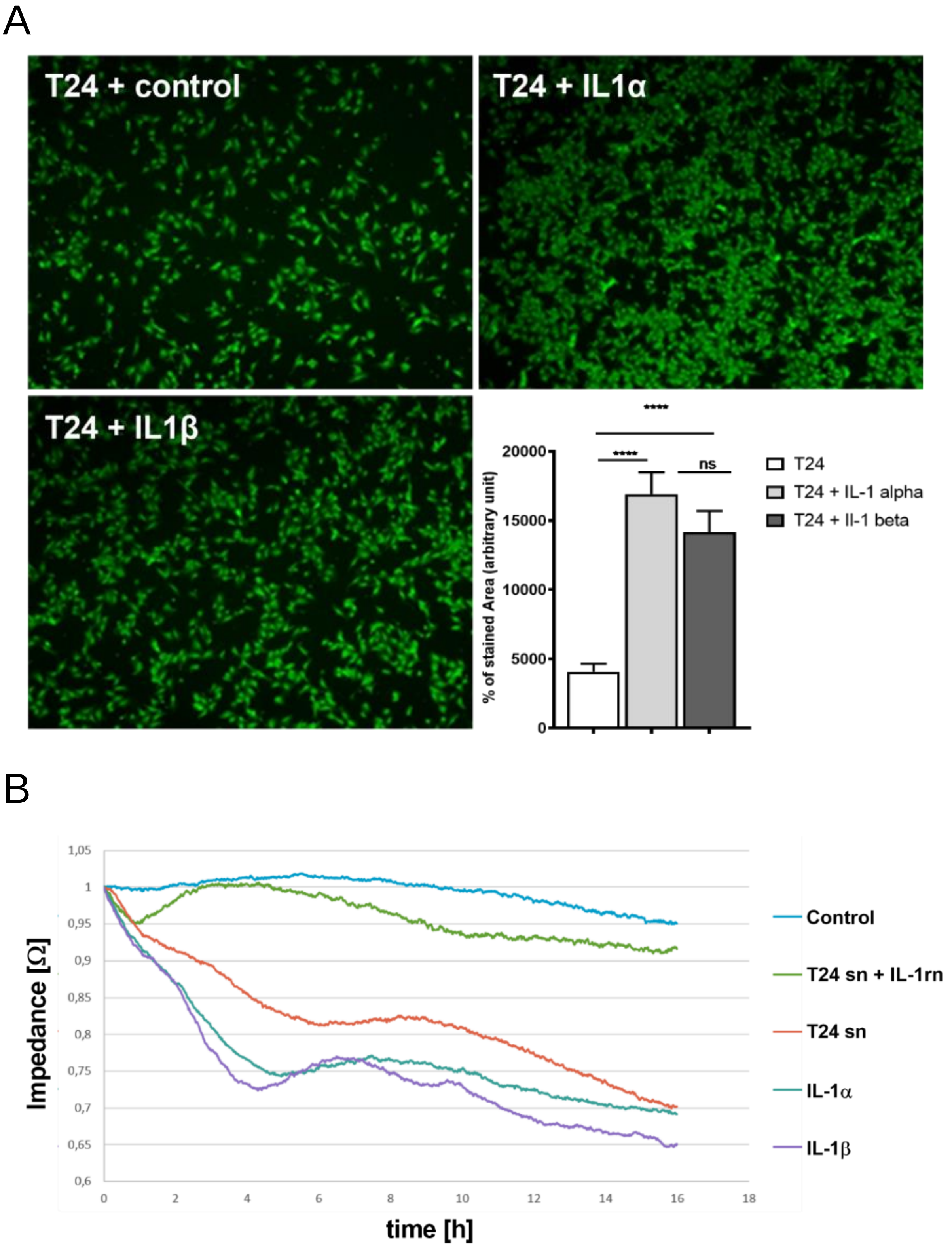

Supplemental Figure S1. IL-1rn impairs IL1α and -β induced migration of T24 tumor cells. **(A)** Modified Boyden Chamber experiment (see Materials and Methods on page 12 for experimental details). T24 tumor cells migrate through a transwell coated with an endothelial cell (Human Umbilical Vein Endothelial Cells / HUVECs) monolayer. Addition of IL1A or IL1B significantly enhances transendothelial migration. (**B)** Impedance sensing of a stable HUVEC monolayer shows barrier breakdown due to incubation with tumor cell secretome. IL1A and IL1B alone were also sufficient to impair the electrical impedance while IL1rn could largely reverse this effect.
